# Latent tuberculosis infection in patients with psoriasis using immunobiologicals: an observational study^☆^

**DOI:** 10.1016/j.abd.2024.09.011

**Published:** 2025-04-16

**Authors:** Mariana Baptista Angeluci, Marilda Aparecida Milanez Morgado de Abreu, Ana Cláudia Cavalcante Esposito Lemos, Eduardo Vinicius Mendes Roncada, Cristhiana Kise Saito, Felipe Puga Barbosa

**Affiliations:** Department of Dermatology, Hospital Regional de Presidente Prudente, Universidade do Oeste Paulista, Presidente Prudente, SP, Brazil

Dear Editor,

Immunobiologicals are an increasingly important therapy in the context of inflammatory diseases, mainly in dermatology, with numerous approved medications.^1^, ^2^ However, the use of these agents is associated with immunosuppressant effects and, consequently, increases the risk of reactivation of latent infections, such as tuberculosis (TB).^2^

Recent guidelines recommend carrying out TB screening before starting treatment with any immunobiological agent, using the tuberculin skin test (TST) or interferon gama release assay (IGRA), as well as being repeated annually in high-risk patients. For patients who are not considered at high risk, this screening is particularly important for those receiving anti-tumor necrosis factor (TNF) agents.^3^ Despite the recommendation, this is not done frequently in practice, so the objective of the present study was to evaluate the prevalence of TB infection (assessed via TST) in patients receiving immunobiological therapy for psoriasis.

This study was carried out at the Regional Hospital of Presidente Prudente, São Paulo, Brazil, and was started only after the project was approved by the Research Ethics Committee of the University of West Paulista (Protocol: 65581122.0.0000.5515 – December 18, 2023) and only those who signed an informed consent form were enrolled. Patients aged 18 years or older who were diagnosed with psoriasis, who were receiving immunobiological agents for at least 6 months, and who had undergone TST before the start of treatment were included. Patients with immunosuppressive diseases or a history of previous TB were excluded.

The TST result was considered negative when there was no induration formation or if it was smaller than 5 mm. The presence of an induration with a diameter greater than or equal to 5 mm when the previous TST was zero or an increase of 10 mm or more from the value of the previous TST, if it was positive, was considered to indicate a positive result. These values were used on the basis of the guidelines of the Brazilian Ministry of Health.^4^, ^5^ Although IGRA is a test with greater specificity in diagnosing latent tuberculosis infection (LTBI), it has a high cost and is currently available in Brazil through the Unified Health System (SUS) only for certain selected conditions.^6^

A total of 85 patients with psoriasis were evaluated and the immunobiologicals used were anti-Interleukin (IL) agents (77.6%), with 25 patients using ustekinumab, 30 using secukinumab and 11 using risankizumab, and anti-TNF agents (22.4%), with 18 patients using adalimumab and 1 patient using etanercept. In 46 patients (63%) there was concomitant use of methotrexate. Table 1 presents demographic and clinical characteristics of psoriasis patients. Table 2 presents the data on the characteristics of the patients with positive results for the second TST. No statistically significant associations were observed for any of the characteristics evaluated.

**Table 1:** Demographic and clinical characteristics of psoriasis patients using immunobiologicals.

Characteristics	Options	n (%)
Sex	Male	44 (51.8%)
	Female	41 (48.2%)
Age	Minimum	25
	Maximum	86
	Mean ± standard deviation	54.2 ± 14.1
Skin color	White	53 (67.1%)
	Brown	21 (26.6%)
	Black	4 (5.1%)
	Yellow	1 (1.3%)
Time of psoriasis diagnosis in years	Minimum	3
	Maximum	43
	Median (IIQ)	9 (5.7–12.0)
Total time of use of immunobiologicals in months	Minimum	11
	Maximum	170
	Median (IIQ)	42 (26–60)
Immunobiological in use	anti-IL	66 (77.6%)
	anti-TNF	19 (22.4%)
Concomitant use of methotrexate	Yes	46 (63.0%)
	No	39 (37.0%)
Previous use of anti-TNF	Yes	32 (37.6%)
	No	53 (62.4%)
First TST	Negative	74 (87.1%)
	Positive	11 (12.9%)
2nd TST was positive or increased > 10 mm	Yes	10 (11.8%)
	No	75 (88.2%)
Time elapsed between TSTs in months	Minimum	9
	Maximum	156

TST, Tuberculin Skin Test; IIQ, Interquartile Range.

Note: Values presented in the form of simple frequencies and percentages for categorical variables, and for quantitative variables as mean ± standard deviation for those that presented normality and median (IIQ) for those that did not present normality. Percentages and summary measures were calculated for the total valid responses for each variable.

**Table 2:** Association between the positive result of the 2^nd^ tuberculin skin test or an increase greater than 10 mm with the demographic and clinical characteristics of psoriasis patients using immunobiologicals.

Characteristics		2^nd^ TST positive or increased > 10 mm		p-value
		Yes	No	
Sex	Male	5 (50.0%)	39 (52.0%)	0.905
	Female	5 (50.0%)	36 (48.0%)	
Age		54.3 ± 16.1	54.2 ± 13.9	0.986
Skin color	White	6 (75.0%)	47 (66.2%)	1.0
	Brown	2 (25.0%)	19 (26.8%)	
	Black	0 (0.0%)	4 (5.6%)	
	Yellow	0 (0.0%)	1 (1.4%)	
Time of psoriasis diagnosis in years		8.5 (6.2–13.7)	9.5 (5.0–12.0)	0.629
Total time of use of immunobiologicals in months		26 (22.7–50.0)	44 (30.5–60.0)	0.238
Immunobiological in use	anti-IL	9 (90.0%)	57 (76.0%)	0.445
	anti-TNF	1 (10.0%)	18 (24.0%)	
Concomitant use of methotrexate	Yes	5 (50.0%)	41 (65.1%)	0.359
	No	5 (50.0%)	22 (34.9%)	
Previous use of anti-TNF	Yes	4 (40.0%)	28 (37.3%)	1.0
	No	6 (60.0%)	47 (62.7%)	
First TST	Negative	8 (80.0%)	66 (88.0%)	0.611
	Positive	2 (20.0%)	9 (12.0%)	
Time elapsed between TSTs in months		27.5 (26.0–47.2)	41 (21.0–55.5)	0.843

TST, Tuberculin Skin Test.

Note: Values presented as Mean ± Standard Deviation for the age variable, median (IIQ) for the other quantitative characteristics, and simple frequencies (%) for the categorical variables. P-values referring to the Chi-Square or Fisher's exact test (when appropriate) for categorical variables, the Mann-Whitney test for comparison between medians, and T-Student test for age.

The second TST was positive, with a diagnosis of LTBI being made in 10 patients (11.7%), of whom 9 used an anti-IL agent (5 secukinumab, 3 ustekinumab, and 1 risankizumab) and only 1 patient used an anti-TNF agent (adalimumab). Despite the higher prevalence of LTBI in patients using anti-ILs, there was no statistical significance, probably because the vast majority of patients included in the study were using anti-ILs. This result is similar to a study also carried out in Brazil with rheumatological patients using anti-TNF, where the TST became positive in 12.3% of patients.^7^

Of the 10 patients who were positive on the second TST, 8 were negative on the first TST, suggesting that they may have acquired the infection while using immunobiological agents. This is probably due to the fact that the region where the study was carried out has a high prevalence rate of TB. Among the 9 positive patients who used anti-ILs, 4 had previously used an anti-TNF agent (1 infliximab and 3 adalimumab), which makes it impossible to infer which medication the positivity would be implicated in or whether it occurred due to the sum of the effects of the two therapies. It is important to highlight that none of the patients developed active TB.

The tuberculin skin test has certain limitations and may present false-positive results due to BCG vaccination and exposure to nontuberculous mycobacteria. The effects of BCG vaccination on TST results are smaller after 15 years. Therefore, a strong positive test, with an induration equal to or greater than 15 mm, has a greater chance of indicating TB infection than occurring as a result of the vaccine.^8^ As the BCG vaccine in Brazil is administered in the first year of life and the patients included in this study were over 18 years old, TST observed positivity has to be understood as a real latent TB infection. Furthermore, most patients had results greater than 15 mm, which also strengthened that the test positivity was due to real infection. False negatives can also occur due to problems with the reagent, inaccurate administration or interpretation of the test, or tuberculin anergy in children and immunocompromised patients, including those who used methotrexate.^5^ However, these possibilities were considered remote, as the PPD kits were used within their validity period and kept in a suitable environment, the test was carried out by the same trained professional, and the only cause of immunosuppression in these patients was the previous or current use of methotrexate and immunobiologicals.

A significant limitation of this study is that it was carried out in a single institution and with a small sample. Other limitations are that some patients concomitantly used methotrexate or previously used anti-TNF agents, all of which may influence the LTBI outcome.

In conclusion, we demonstrated the development of LTBI in an important proportion of patients receiving immunobiologicals. Furthermore, no patient developed active TB, which may demonstrate the possible safety of this group of immunobiologicals. We emphasize the need for periodic assessment of TB during immunobiological treatment, especially in endemic countries, such as those in Latin America, to carry out early detection and avoid active disease.

## Financial support

None declared.

## Authors’ contributions

Mariana Baptista Angeluci: Design and planning of the study; collection, analysis and interpretation of data; drafting and editing of the manuscript; critical review of the literature; approval of the final version of the manuscript.

Marilda Aparecida Milanez Morgado de Abreu: Design and planning of the study; drafting and editing of the manuscript; collection, analysis and interpretation of data; effective participation in research orientation; approval of the final version of the manuscript.

Ana Cláudia Cavalcante Esposito Lemos: Design and planning of the study; effective participation in research orientation; approval of the final version of the manuscript.

Eduardo Vinicius Mendes Roncada: Collection of data; effective participation in research orientation; approval of the final version of the manuscript.

Cristhiana Kise Saito: Analysis and interpretation of data; drafting and editing of the manuscript; approval of the final version of the manuscript.

Felipe Puga Barbosa: Collection of data; statistical analysis; approval of the final version of the manuscript.

## Conflicts of interest

None declared.
